# Dengue Virus Capsid Protein Binds Core Histones and Inhibits Nucleosome Formation in Human Liver Cells

**DOI:** 10.1371/journal.pone.0024365

**Published:** 2011-09-01

**Authors:** Tonya M. Colpitts, Sebastian Barthel, Penghua Wang, Erol Fikrig

**Affiliations:** 1 Section of Infectious Diseases, Department of Internal Medicine, Yale University School of Medicine, New Haven, Connecticut, United States of America; 2 Howard Hughes Medical Institute, Yale University School of Medicine, New Haven, Connecticut, United States of America; 3 Interfaculty Institute of Biochemistry, University of Tuebingen, Germany; Duke-National University of Singapore Graduate Medical School, Singapore

## Abstract

Dengue virus (DENV) is a member of the *Flaviviridae* and a globally (re)emerging pathogen that causes serious human disease. There is no specific antiviral or vaccine for dengue virus infection. Flavivirus capsid (C) is a structural protein responsible for gathering the viral RNA into a nucleocapsid that forms the core of a mature virus particle. Flaviviral replication is known to occur in the cytoplasm yet a large portion of capsid protein localizes to the nucleus during infection. The reasons for the nuclear presences of capsid are not completely understood. Here, we expressed mature DENV C in a tandem affinity purification assay to identify potential binding partners in human liver cells. DENV C targeted the four core histones, H2A, H2B, H3 and H4. DENV C bound recombinant histones in solution and colocalized with histones in the nucleus and cytoplasm of liver cells during DENV infection. We show that DENV C acts as a histone mimic, forming heterodimers with core histones, binding DNA and disrupting nucleosome formation. We also demonstrate that DENV infection increases the amounts of core histones in livers cells, which may be a cellular response to C binding away the histone proteins. Infection with DENV additionally alters levels of H2A phosphorylation in a time-dependent manner. The interactions of C and histones add an interesting new role for the presence of C in the nucleus during DENV infection.

## Introduction

Dengue virus (DENV, serotypes 1–4) is an enveloped, single-stranded RNA (ssRNA) virus and a member of the mosquito-borne flaviviruses, which also include West Nile virus (WNV) and Japanese encephalitis virus (JEV) [1]. DENV is a serious human pathogen causing life-threatening diseases such as dengue fever (DF) and dengue hemorrhagic fever (DHF), predominantly in (sub)tropical regions worldwide. There are an estimated 100 million cases of DENV infection per year, with over 500,000 cases of potentially fatal dengue hemorrhagic fever (DHF). Currently, there is no specific antiviral available to treat DENV infection and vaccine efforts have been hindered due to safety concerns [2], [3]. A deeper understanding of the molecular interactions of DENV proteins with host cellular factors during infection is needed to develop new strategies to prevent and treat dengue virus infection.

The flaviviral RNA genome encodes a single precursor polyprotein which is cleaved co- and posttranslationally into three structural (C, prM/M and E) and seven non-structural (NS) proteins (NS1, NS2A, NS2B, NS3, NS4A, NS4B, NS5) by viral and cellular proteases [4]. Mature DENV capsid protein (C) is a small, highly basic 12-kDa protein and an essential factor during virion assembly. DENV C is involved in the proper encapsidation of the RNA genome, resulting in a spherical nucleocapsid with a single copy of the ssRNA molecule [5]. The solution structure of DENV C was previously solved and reveals a dimeric alpha-helical protein [6], [7]. Though flaviviral replication takes place in the cytoplasm and the perinuclear region of endoplasmic reticulum [8], a proportion of C protein is known to translocate to the nucleus and nucleoli of infected cells [9] via three nuclear localization signals (NLS) [10]. Additionally, flaviviral C has been shown to bind and interact with several nuclear proteins, including alpha-importin to enter the nucleus [11], Daxx protein to induce Fas-mediated apoptosis [12], HDM2 to regulate p53-dependent apoptosis [13] and a nuclear protein phosphatase 2A inhibitor [14]. However, the biological significance for the nuclear presence of flaviviral C during infection is still not understood in its entirety.

In this study, we sought to identify and investigate other host cell nuclear proteins that bind DENV C using a tandem-affinity purification (TAP) assay and the human liver cell line Huh7. The TAP method enables the purification of interacting protein complexes in live cells, under near-to-physiological conditions. TAP has been used to identify a number of mammalian cellular proteins that bind viral proteins, including the influenza RNA polymerase, human papillomavirus E1 helicase and the Epstein-Barr virus (EBV) nuclear antigen 5 [15], [16], [17]. We chose to use Huh7 cells as the liver is known to be involved during human infection with dengue virus [18], [19]. Using the TAP assay, we show that the nuclear histone proteins, H2A, H2B, H3 and H4, are specific targets of DENV serotype 2 (DEN2) C. Histone proteins are highly conserved and alkaline proteins found in all eukaryotic cells, where they facilitate the compaction of chromosomal DNA into its chromatin structure [20]. A histone octameric core, composed of two copies each of H2A, H2B, H3 and H4, wrapped with 146 bp of DNA, forms the nucleosome [21]. Histones also fulfill important cellular functions in gene regulation and chromatin accessibility [22]. In the present study, we analyze the interaction of DENV C with the four cellular core histones (H2A, H2B, H3, H4) and examine the molecular impact of capsid-histone binding during DENV infection.

## Results

### DENV C binds host cell histones in liver cells and in solution

**Figure 1:**
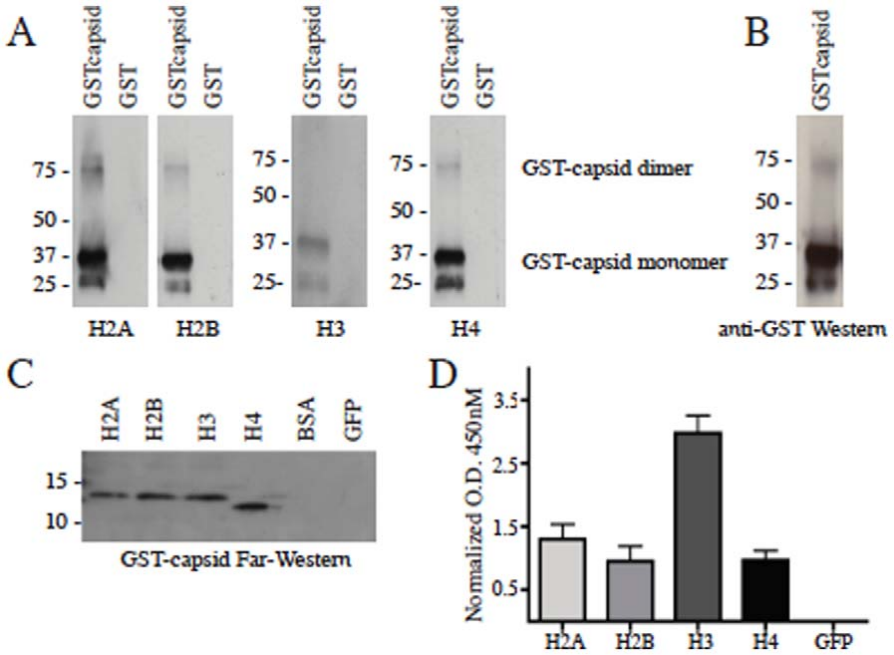
DENV C binds mammalian core histone proteins. (A) Far Western blot. Purified GST-tagged DEN2 C and GST alone were run on a 4–12% SDS-PAGE gel and transferred to nitrocellulose. The membrane was incubated with each of the core histones (H2A, H2B, H3, H4) for 1 h at RT and detected using rabbit polyclonal antibodies against the histones. (B) Western blot of GST-tagged DEN2 C using an antibody against GST for detection. (C) Far Western assay done with recombinant histone proteins and probed with GST-tagged DENV C in the same manner as (A). Control proteins for specificity are BSA and GFP (far right lanes). (D) Graph of ELISA measuring levels of histones or GFP that bound GST-tagged DEN2 C, normalized to levels that bound GST alone.

To identify DENV C binding partners, the mature DEN2 C protein was cloned into the NTAP tagged expression vector and expressed in Huh-7 human liver cells. Liver cells were used in our assay as the liver is a main target in natural dengue infection. Expression of tagged-DENV C localized mainly to the cell nucleus (Figure S1). Using the tandem affinity purification (TAP) assay, several mammalian proteins were identified as targets of dengue virus capsid protein. All four core histones (H2A, H2B, H3 and H4) were pulled out of the cell lysates by DEN2 C and were identified with high percent coverage and low expectation values (Table 1). None of the histone proteins were pulled out by control vector or by tagged-green fluorescent protein. We chose the interaction between C and histones for further investigation, as histones are predominantly found in the nucleus of cells and may be a nonstructural target of C nuclear localization during DENV infection. Far Western assays using either recombinant C protein with histones as probes (Figure 1A) or histone proteins with recombinant GST-tagged C as the probe (Figure 1C) confirmed the binding between C and core histones H2A, H2B, H3 and H4. Histone proteins did not bind GST alone and C did not bind to either BSA or GFP, which were run as controls (Figure 1A and 1C). Figure 1B shows GST-tagged DEN2 C in a Western blot, detected by an antibody against GST, for reference. Finally, an ELISA was done to further confirm the binding between DEN2 C and the core histones H2A, H2B, H3 and H4 (Figure 1D). DENV C bound all four histones but not GFP protein and histones bound C at levels 0.75 to 2.75 higher than GST alone.

**Table 1:** Mammalian core histones were identified as DEN2 C binding partners.

Score	Expectation	Protein	% Coverage
372	5.00E-94	Histone 2A, type 1B/E	43.8
335	2.50E-29	Histone 4	60.2
313	4.00E-27	Histone 2B, type 1-L	48.4
312	5.30E-27	Histone 2A, type 2A	43.8
64	0.033	Histone 3.2	24.3

The 100 amino acid mature DEN2 C protein fused to streptavidin binding protein and calmodulin binding protein (CBP) tags was expressed in Huh7 liver cells and interactions identified using a tandem affinity purification assay. Proteins that bound tagged DEN2 C were isolated and identified by LC-MS/MS spectrometry.

### DENV C associates with host cell histones during overexpression

**Figure 2:**
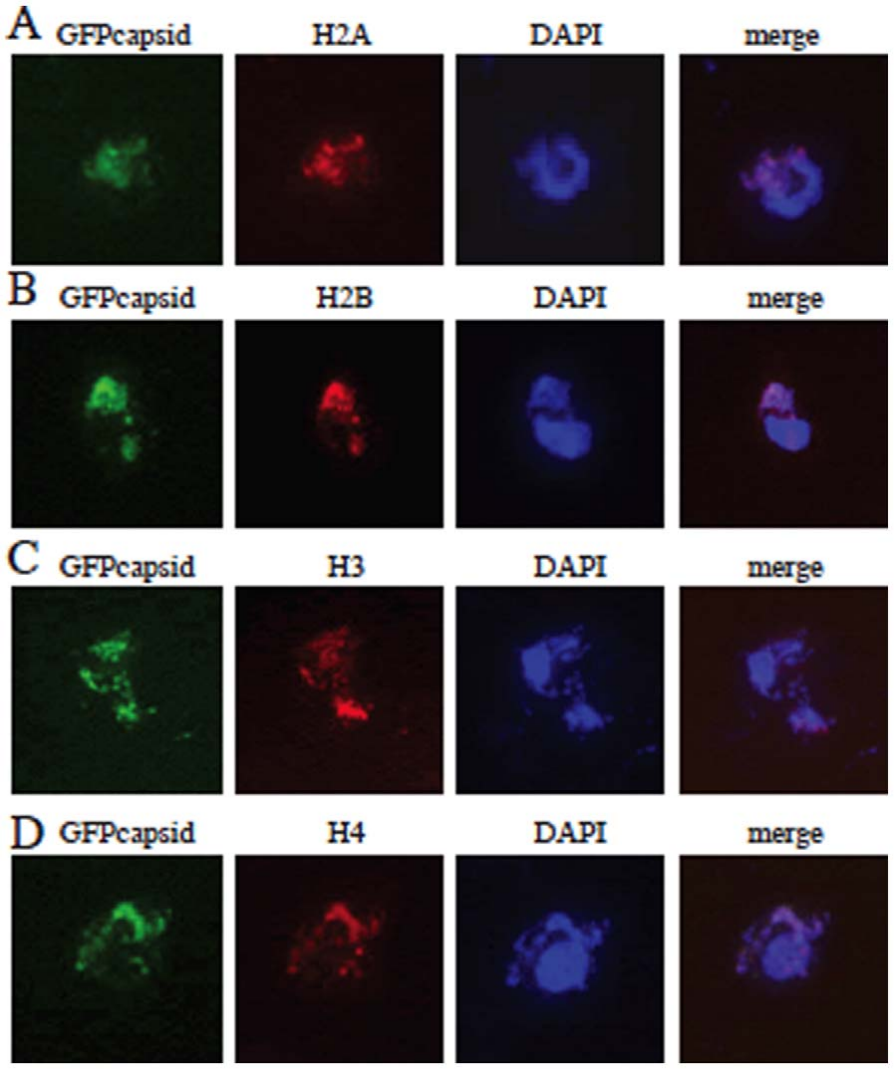
DENV C colocalizes with histones in Huh7 liver cells. DEN2 C colocalized with H2A (A), H2B (B), H3 (C) and H4 (D) in Huh7 cells. Cells were transfected with GFP-DEN2 C and fixed in 4% para-formaldehyde 48 h post-transfection. Cells were stained with antibodies against histones and a TRITC secondary antibody. Cells were counterstained with DAPI to visualize the nucleus. GFP-DEN2 C expression is green, histone staining is red and DAPI is blue.

Although histone-C binding was confirmed both in solution and infected cell lysates, it was important to look at the interaction in a physiologically relevant cellular environment. To examine the association of DENV C with histones in live cells, the mature DEN2 C protein was cloned into a green fluorescent protein (GFP) fusion vector, NGFP-TOPO, and this fusion protein expressed in the human liver cells. We examined the cellular location of both DEN2 C and cellular histone proteins. The expression of DEN2 C was mainly localized to the nucleus of cells and altered cell morphology when compared to expression of GFP alone (Figure S2). Cell viability was not significantly altered, both by DAPI visualization and by trypan blue exclusion test (at 24 h post-transfection, cells were over 80% viable). At 24 h post-transfection, DEN2 C colocalized with all four core histones, mainly in the nucleus of the cells. A portion of colocalized protein was also found in the cytoplasm, both with and without DNA, especially when looking at the association of DENV C and H3 (Figure 2A–D). Distinct foci can be seen that represent an accumulation of both core histones and DEN2 C.

### DENV C forms dimers with histones in solution

**Figure 3:**
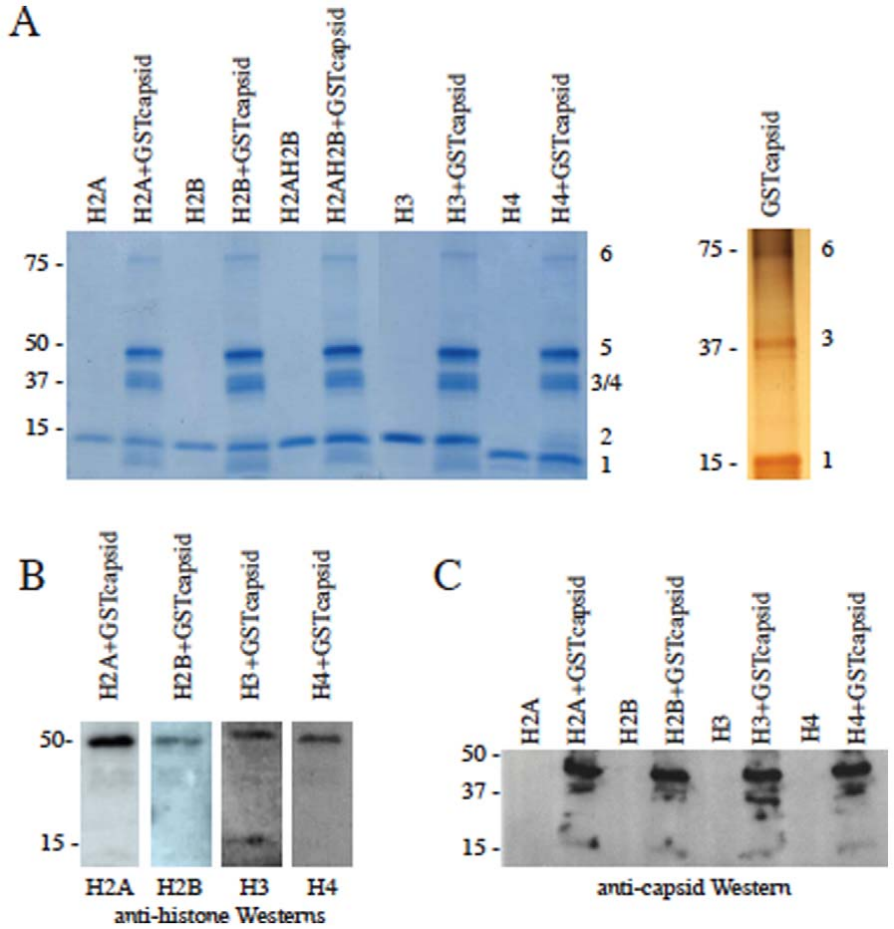
DENV C forms dimers with core histones in solution. (A) Purified GST-tagged DEN2 C and recominant histone proteins were incubated in solution for 1 h at 37°C. Proteins were separated on 4–12% SDS-PAGE gel and stained with Coomassie blue (left panel) and silver stain (GST-capsid only; right panel). Numbers at right of gel images indicate the following bands: 1-capsid monomer, 2-histone monomer, 3-GST-capsid monomer, 4-histone dimer, 5-GST-capsid/histone dimer, 6-GST-capsid dimer. (B) Histones and (C) DENV C are detected in presumed heterodimers from (A) using antibodies against histones and DEN2 capsid in Western blot assays.

Histones are known to form dimers through interactions between hydrophobic amino acids in their second and third alpha helices [23]. As DEN2 C is also an alphahelical protein with the ability to self-dimerize and contains a number of hydrophobic residues in its second alpha helix [6], [24] (Figure S3), we examined whether histones and DEN2 C could form heterodimers in solution. The incubation of recominbant core histone proteins with GST-tagged C did result in the formation of dimers, which were resistant to both heat and SDS-denaturation. The histone-C dimers in solution were run on an SDS-PAGE gel and stained with Coomassie for visualization (Figure 3A). The GST-tagged C is 38 kD and both histones and C are 11–14 kD, therefore homodimers of either histones or C are expected to run at 22–28 kD, which is visible on the gel around the same region as GST-C monomers. A histone-C heterodimer would be expected to run at about 40 kD, which is visible as a dark blue band in the gel (Figure 3A). To confirm the presence of both DEN2 C and histones in this presumed dimer band, antibodies against C and against each of the histones were used in Western blots made with corresponding gels (Figure 3B–C). The addition of both deoxyribonuclease (Dnase I) and ribonuclease (Rnase) to the solution of histones and DEN2 C did not prevent the heterodimerization. This indicates that the binding is not solely due to nucleic acid interaction and that C and histones are able to associate in the absence of both DNA and RNA.

### DENV C binds DNA as well as host cell histones and does not disrupt histone-DNA binding

**Figure 4:**
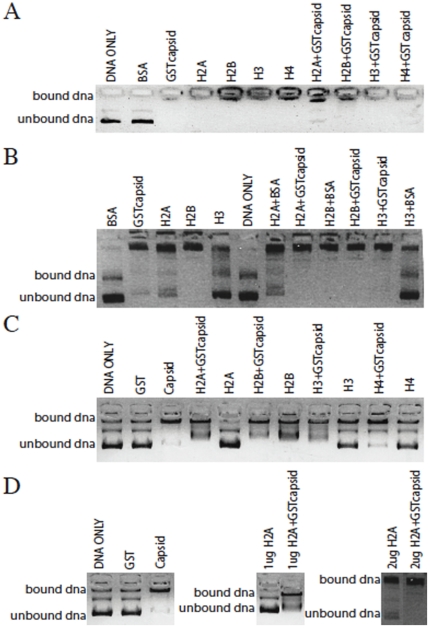
DENV C binds DNA and does not disrupt histone-DNA binding. (A) 5 µg or (B) 2 µg of BSA or histone proteins H2A, H2B, H3 and H4 were incubated with 300 ng plasmid DNA, with and without 10 µg GST-tagged DENV C, for 1 h at 37°C. (C) 1 µg of GST or H2A, H2B, H3 and H4 were incubated with 300 ng plasmid DNA, with and without 10 µg GST-tagged DENV C, for 1 h at 37°C. (D) 10 µg GST or 10 µg untagged DENV C was incubated with 300 ng plasmid DNA (left panel), 1 µg (middle panel) or 2 µg (right panel) of H2A was incubated with 300 ng plasmid DNA, both with and without 10 µg GST-tagged DENV C. Solutions were run on 1% agarose gel with ethidium bromide to visualize DNA migration. Bound/unbound DNA is indicated.

Given that DENV C dimerized with host cell histones and the alpha-helical structure of the two proteins is almost identical, we next wanted to investigate whether C could disrupt histone-DNA binding. To evaluate this, we used a DNA-binding assay. Recombinant histones with and without DEN2 C, as well as DEN2 C alone, were incubated with plasmid DNA and the solution was run on agarose gel with ethidium bromide to visualize DNA bands. All four core histones formed a histone-DNA complex when incubated with plasmid DNA. We also found that the addition of C to the histone-DNA solution did not disrupt DNA binding to any of the four core histones. In addition, C alone was able to bind DNA without the presence of histones (Figure 4A). In fact, C appeared to bind DNA better than any of the core histones in this assay. We next titrated the amount of histone protein to measure if C could compensate for the lack of histones. When the amount of histone protein in solution was reduced from 5 µg to 2 µg, not all of the DNA plasmid was bound. In this case, the addition of GST-tagged DEN2 C protein increased the binding of DNA, indicating that either C alone or C bound to histones will directly bind DNA (Figure 4B). GST protein alone was not able to bind DNA and 10 µg of untagged DEN2 C bound DNA as well as GST-tagged DEN2 C (Figure 4C). Figure 4D shows a summary of the data using H2A, showing histone-DNA complexes at low and high protein amounts and that the addition of DEN2 C increases DNA binding at both concentrations. These results suggest that DEN2 C is likely not targeting the DNA-binding site of core histones, as the histone-DNA binding is not disrupted by the presence of C. This also indicates that DEN2 C may be acting as a histone mimic in the cell not only by forming dimers with histones but also by binding DNA itself, either alone or in oligomeric form.

### DENV C expression disrupts nucleosome formation in live cells

**Figure 5:**
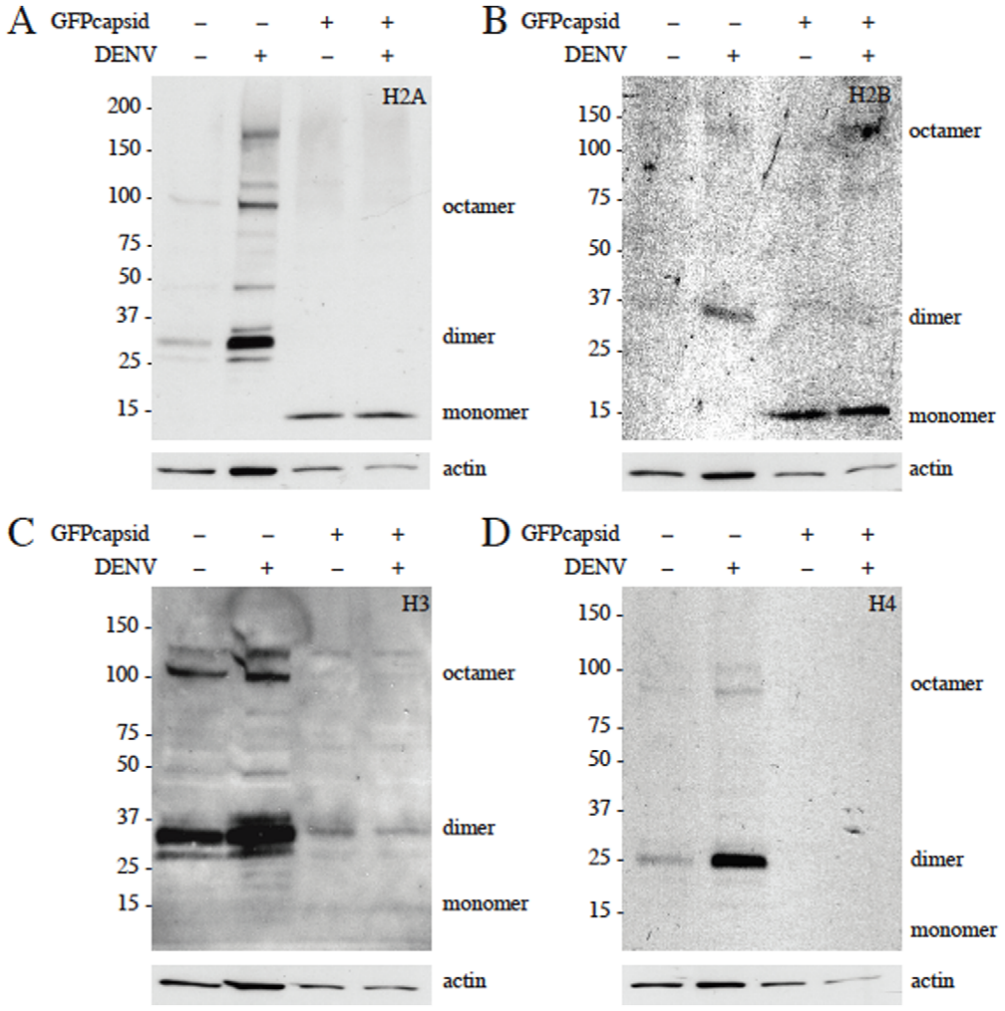
DENV expression disrupts histone oligomerization. Huh7 cells were transfected with DEN2 C and/or infected with DEN2 24 h post-transfection. Cells were lysed 24 h post-infection (48 h post-transfection) and lysates were run on 4–12% SDS-PAGE gel. Gels were used in a Western blotting assay with antibodies against histones H2A (A), H2B (B), H3 (C) and H4 (D); monomers, dimers and octamers are indicated. Gels were stripped and reprobed with an antibody against actin as a protein loading control. The same amount of protein was loaded in each lane for each gel as a control for expression.

As DENV C was found to bind, colocalize and form dimers with the four core histones, H2A, H2B, H3 and H4 (Figures 1, 2, 3), we next wanted to explore the cellular effects of the interaction. To evaluate the functional significance of this association, DENV C was expressed in liver cells and the cell lysates were isolated to examine histone expression and oligomer formation. Cell lysates were run on an SDS-PAGE gel and histone proteins were transferred and detected via Western blot using antibodies against histones. The same amount of protein was loaded in each well to normalize the data. In untransfected and DEN2-infected cells, histones formed dimers, tetramers and octamers that were resistant to heat and SDS denaturation (Figure 5). The histone oligomers disappeared during expression of DEN2 C protein, with or without DEN2 infection of the cells. Overexpression of DEN2 C caused an accumulation of histone H2A and H2B monomers (Figures 5A–B), while expression disrupted the formation of H3 and H4 dimers without monomer build-up (Figures 5C–D). The overexpression of C seemed to cause a reduction in total histone protein when compared to DEN2 infection and infection alone appeared to increase the amount of total histones. The overexpression of C also decreased the levels of actin in the cells, which can be partially attributed to cell death, a known result of flavivirus C expression. We thought that the dimerization of DEN2 C and host cell histones as well as the disruption of histone oligomerization could impact the numerous post-translational modifications (PTMs) that occur on histones, which could affect cellular functions from regulation of gene expression to DNA damage repair. We looked at the methylation of H3 during DENV infection both with and without expression of DENV C to investigate one facet of this complex issue. We found that, although dimerization of H3 was greater during DEN2 infection and eliminated during overexpression of DEN2 C, there was no apparent difference in dimethylation of the H3 dimers during DEN2 infection (Figure S4).

### DENV C colocalizes with histone proteins during DENV infection

**Figure 6:**
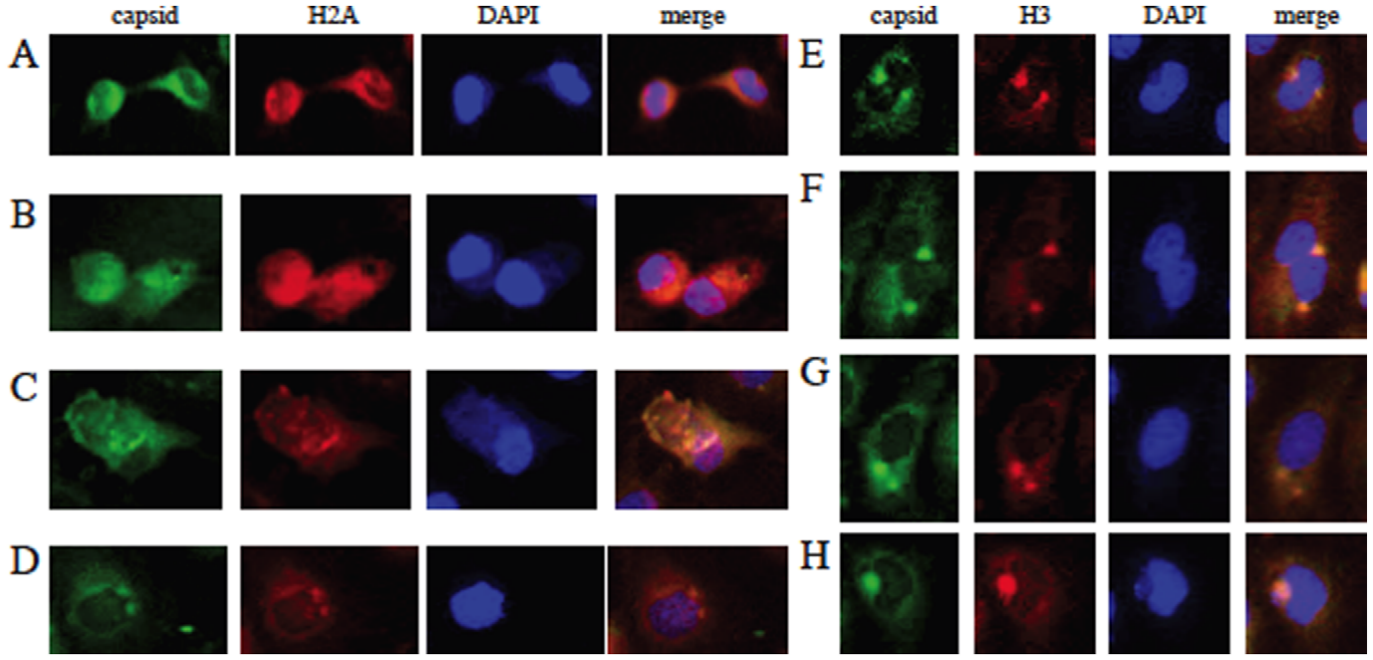
DENV C colocalizes with histones during DENV infection. Huh7 cells were infected with DEN1 (A and E), DEN2 (B and F), DEN3 (C and G) and DEN4 (D and H) and fixed 24 h post-infection with 4% para-formaldehyde. Cells were stained with antibodies against histone 2A (A–D) and histone 3 (E–H) with a TRITC secondary antibody as well as mouse immune serum against DENV C protein (A–H) with a FITC secondary antibody. Cells were counterstained with DAPI to visualize the nucleus. DENV C expression is green, histone staining is red and DAPI is blue.

**Figure 7:**
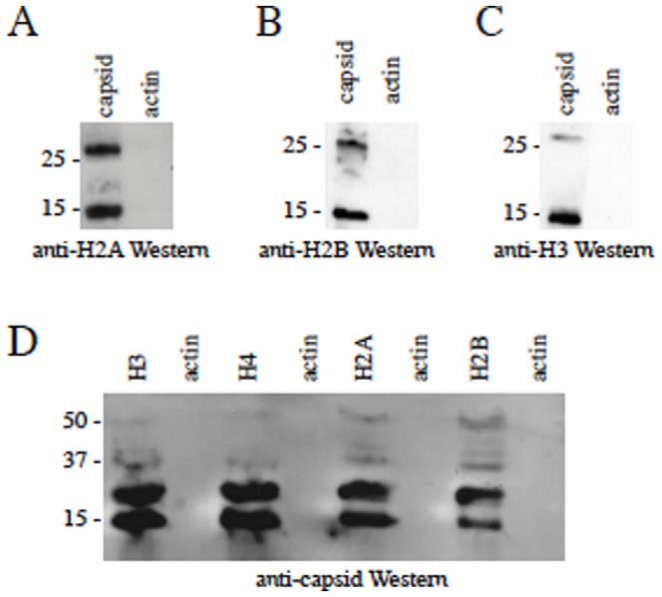
DENV C binds core histones during DENV infection. (A,B,C) DEN2 C and core histones were co-immunoprecipitated during DEN2 infection of Huh-7 liver cells. Cells were lysed 24 h post-infection with DEN2 at moi 0.1. DENV C was immunoprecipitated from the cell lysate using anti-capsid serum, run on a 4–12% SDS-PAGE gel and transferred to nitrocellulose. The membrane was probed with antibodies against H2A (A), H2B (B) and H3 (C). (D) The H2A, H2B and H3 proteins were separately immunoprecipitated from the lysate and the solutions were run on a 4–12% SDS-PAGE gel and transferred to nitrocellulose. The membrane was probed with mouse immune serum against DEN2 C protein. Immunoprecipitate using beta-actin antibody was used as control in A–D.

Having established that DENV C binds and associates with core histones both in solution and during overexpression, we next examined the cellular distribution of both C and histones during DENV infection of liver cells. The C protein from all four serotypes of DENV, (DEN1, DEN2, DEN3 and DEN4), was seen to associate with all four core histones during infection. Figure 6 shows the colocalization of DEN1-4 with H2A and H3. The distribution of H2A is both nuclear and cytoplasmic and strongly correlates with the distribution of DENV C protein. The localization of H3 was mostly in distict foci in the perinuclear region and we were able to find C from all four serotypes of DENV colocalizing to the same areas. This data demonstrates that C and core histones associate in live cells both with and without DENV infection. We next examined whether this association represented actual binding between the C and histone proteins during DENV infection. To examine this, co-immunoprecipitation assays were done on DEN2-infected Huh-7 cell lysates. We were able to detect H2A, H2B and H3 in solution precipitated from infected cell lysates using mouse immune serum generated in our laboratory against DEN2 C protein (Figure 7A–C). The histone antibodies did not bind to lysates immunoprecipitated using an antibody against beta-actin protein. Unfortunately, we were not able to detect H4 in the lysates precipitated using the anti-C mouse immune serum. We were also able to detect DEN2 C protein in solution precipitated from infected cell lysates using antibodies against all four core histones (Figure 7D). DENV C was not detected in solution precipitated with antibodies against beta-actin.

### DENV infection alters total protein and phosphorylation levels of H2A over time

**Figure 8:**
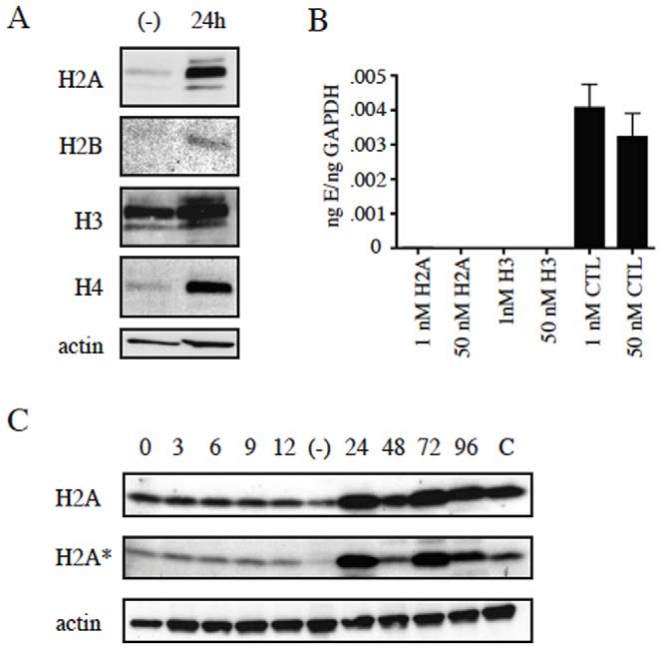
DENV infection alters levels of core histones. A. Huh7 cells were infected with DEN2 and lysed 24 h post-infection. Uninfected cells were also incubated as control. Cell lysates were run on 4–12% SDS-PAGE gel and gels were used in a Western blotting assay with antibodies against histones H2A, H2B, H3 and H4, as well as actin for loading control. B. Histone siRNA reduces DENV infection. Huh7 cells were transfected with siRNA targeting either H2A or H3 and infected with DEN2 48h post-transfection. Transfected and uninfected cells were also incubated as control. RNA was isolated from cells and cDNA used in a real-time qPCR reaction to measure dengue infection. Infection is indicated as copy number of envelope gene (E)/copy number GAPDH. C. DENV infection alters H2A phosphorylation in a time-dependent manner. Huh7 cells were either transfected with GFP-DEN2 and lysed 24 h post-transfection or infected with DEN2 and lysed post-infection. Cell lysates were run on 4–12% SDS-PAGE gel and gels were used in a Western blotting assay with antibodies against H2A, phosphorylated H2a (H2A*) and actin as loading control. 0–96 indicates hour post-infection, (−) indicates uninfected lysate and C indicates cells transfected with capsid for 24 hours.

Since DENV infection as well as the expression of DENV C alone altered levels of histone oligomerization in cells, we next examined the levels of core histone H2A as well as H2A phosphorylation during DENV infection over time. Huh-7 cells were infected with DEN2, lysed 24 h post-infection and assayed for amounts of histone protein. Infection with DENV increased the levels of all four core histone proteins in the liver cells, especially H2A (Figure 8A). Since infection increased histone levels, it was probably that reduction of histones may impede infection. To test this, siRNAs against H2A and H3 were separately transfected into Huh-7 cells and cells were infected with DENV 48 h post-transfection. The reduction of histone proteins was confirmed using a Western blot assay. When histone protein production was blocked using siRNA, DENV infection was greatly reduced compared to infection of cells transfected with random control siRNA (Figure 8B). The levels of GAPDH mRNA were not significantly altered with the addition of the histone siRNA, indicating that cell health was not the reason for reduced infection. These results suggest that histones H2A and H3 are in fact necessary for productive infection. The enhanced levels of H2A during DENV infection and the reduced infection with elimination of H2A both suggest that this histone likely plays an important role during DENV infection. The phosphorylation of H2A has been shown to be active in the DNA damage response and to recruit proteins involved in DNA repair (28). We chose to look at the levels of phosphorylation of H2A during DENV infection of the cells over time. During the first 12 hours of infection, the cells showed little alteration in the level of H2A phosphorylation (Figure 8C). At 24 h post-infection, phosphorylation of H2A is significantly higher than in uninfected cells. At 48 h post-infection, the blot shows decreased phosphorylation and the levels increase once again at 72 and 96 h post-infection. This indicates that DENV infection not only increases the overall levels of core histones as well as oligomeric forms of histones, but also alters the phosphorylation of H2A in a time-dependent manner.

## Discussion

Several viral proteins target host chromatin and histone proteins to interfere with host gene expression and nucleosome assembly by various mechanisms and for diverse purposes [25]. The influenza virus matrix protein M1 is known to interact with the core histones, though the functional reasons for this remain unclear [26], [27]. *Paramecium bursaria* chlorella virus 1 (PBCV-1) represses host cell gene transcription through H3K27 methylation by a viral SET (vSET) domain containing a histone lysine methyltransferase [28]. Herpes simplex virus type 1 (HSV-1) is known to utilize histones for its own genome during lytic infection and a HSV-1 encoded kinase, US3, has been shown to block histone deacetylation to enable gene expression by inducing the phosporylation of histone deacetylase 1 (HDAC1) [29], [30]. ICP11 protein of white spot syndrome virus (WSSV) acts in a more direct way as a histone-binding DNA mimic to disrupt host nucleosome assembly [31]. In this study, we used a tandem affinity purification assay to identify core histones H2A, H2B, H3 and H4 as host cell targets of DENV C protein. DENV C was shown to bind host cell core histones in a variety of assays and colocalized with all four histones in live human liver cells, both with and without DENV(1–4) infection. The colocalization of DENV C and histones occured both in the nucleus and the cytoplasm, suggesting that in addition to binding histones in the nucleus, DENV C may bind histones before they enter the nucleus and/or pull them into the cytoplasm after binding in the nucleus. We also showed that DENV C disrupted histone dimerization and nucleosome formation and that DENV C formed heterodimers with all four core histones. Given that both DENV C and histones are alphahelical, dimerizing proteins of similar length and secondary structure, the formation of heterodimers between the proteins is not overly surprising. Crystallization studies are underway to further examine the dynamics of the histone-C interaction.

Flaviviral capsid protein has a high content of basic amino acids, which is consistent with its ability to interact with RNA during infection. In the current studies, the enzymatic elimination of DNA and RNA did not prevent capsid-histone dimerization, which indicates that the DENV C-histone binding is not solely due to nucleic acid interactions. DENV C is known to self-dimerize in the absence of nucleic acid [6] and H2A and H2B are known to form dimers during the formation of the nucleosome, presumably before winding the DNA [23], [32]. In a DNA-binding assay, histone-DNA complexes were not disrupted by the addition of DENV C and the viral protein was shown to bind DNA as well as or better than the core histones, which could have additional consequences for the cellular genetic machinery. It will be interesting to perform cross-linking chromatin immunoprecipitation along with pyrosequencing (CLIP/ChIP-Seq) analysis to see if DENV C specifically targets regions of genomic DNA or if there is only general, nonspecific binding. If capsid is specifically binding DNA to alter gene expression, it will also be important to further investigate the role of the interaction in the flaviviral life cycle. We propose a model where DENV C forms heterodimers with native histone proteins through hydrophobic amino acid interactions. DENV C also binds cellular DNA, either in a complex with core histones or alone. In either scenario, the ability of DENV C to bind both histones and cellular DNA as well as to disrupt nucleosome formation likely impedes the genetic mechanisms in the nucleus, which could result in altered gene expression, impaired DNA transcription, greater DNA damage and possibly a shift in translation favoring viral mRNA over cellular mRNA. This is the first time a flaviviral protein has been shown to interact with components of the chromatin and the host cell nucleosome.

Histones are found in the nucleus of the cell and flavivirus capsid often localizes to the nucleus, though the reasons for this are not entirely clear [9]. Other viral proteins that have been found to associate with histones and chromatin are known to function in the nucleus during viral replication. Influenza matrix protein aids in the export of viral ribonucleoprotein to the cytoplasm for virus assembly [33]. The DNA virus proteins likely use chromatin and the cellular nucleosome components for their own replication and gene expression. Flavivirus replication is known to occur in the cytoplasm on cellular membrane structures in the perinuclear region of the cell yet the location of capsid in the nucleus may be linked to RNA replication, at least in the case of JEV C [34], [35]. The flavivirus capsid protein is responsible for both gathering the nucleic acid for virus assembly as well as releasing the viral genome into the cytoplasm during infection. Histone proteins have many functions relating to nucleic acid organization, including winding, unwinding, translation and gene expression. The capsid proteins only bound histones from the core families which specifically wind nucleic acid to form the nucleosome, the main unit of chromatin [20]. With these roles in mind, DENV C may bind histones to aid in some aspect of viral replication or nucleic acid organization. Flaviviral C has been shown to interact with cellular Daxx protein [12], which also interacts with a number of chromatin inhibitory complexes to repress cellular gene transcription [36]. In another viral infection, CMV gene expression is known to be enhanced in Daxx-depleted cells, suggesting that Daxx contributes to the repression of viral replication, perhaps through chromatin interaction [37]. DENV C may be interacting with both histones and Daxx in the nucleus to block the inhibition of viral transcription while rendering cellular nucleosomes disrupted and unable to properly function.

There are several examples of virus infection inducing the activation of DNA damage signaling and other chromatin antiviral activity [38], [39]. HSV-1 promotes phosphorylation of H2AX and chromatin is known to suppress gene activation in EBV through post-translational modifications of histones [40], [41]. DENV C may bind histones to suppress or interfere with possible antiviral activity of chromatin. Further investigation is ongoing to determine whether DENV C interacts with other members of the cellular chromatin complex and what impact these interactions have on infection. Although the addition of DENV C eliminated histone oligomers, DENV infection actually increased the amount of histone protein as well as core histone dimerization. This suggests that viral infection may alter the available functional nucleosomes through DENV C-histone binding and the cell may increase histone production to compensate. We looked at a histone post-transcriptional modification (PTM), H3 dimethylation, to examine the impact of DENV infection. There was no apparent difference in H3 methylation between control and infected cells, though DENV infection did cause an increase in total H3 dimers. If the cell is producing more core histones to compensate for those bound by DENV C, this indicates that not all of them may be correctly routed through the cellular post-transcriptional machinery during infection. There are a great number of histone PTMs that must be examined both during DENV infection and during C overexpression in order to establish what impact DENV C may have on histone function. We also found that phosphorylation of H2A was altered during infection, which could be a cellular response to DNA damage during DENV infection. Alternatively, this could be an indirect downstream effect of other protein interactions in the cell during infection. It has previously been shown that WNV capsid protein binds a phosphatase inhibitor resulting in increased PP2A phosphatase activity (12), which may be important for altering interferon signaling. An investigation into the interactions of DENV proteins with cellular phosphatases would be of interest to further understand the reasons for increased H2A phosphorylation.

In summary, we found that DENV capsid protein binds to and colocalizes with liver cell core histones with and without DENV infection. There was evidence of DENV C-histone heterodimerization in solution and DENV C was able to prevent histone dimerization and nucleosome formation in cells. We found that DENV C is able to bind DNA and does not disrupt histone-DNA binding. In addition, the levels of histone proteins dramatically increased during DENV infection and the presence of the virus altered the levels of H2A phosphorylation over time. These studies add an exciting new role for flavivirus capsid protein in the host cell nucleus during infection, with the possibility that DENV C may target core histones during infection to disrupt normal host cell genetic machinery in favor of viral replication and the viral life cycle.

## Materials and Methods

### Cell lines and cultivation

Huh7 human liver cells (gift of Dr. Brett Lindenbach, Yale University, CT) were used for all transfection and infections studies. The cells were grown at 37°C and 5% CO2 in DMEM supplemented with 10% heat-inactivated fetal bovine serum (Gemini, CA), 1% penicillin-streptomycin and 1% MEM amino acid solution (Sigma, MO).

### DENV C expression plasmid constructs

All plasmids were prepared using DNA miniprep kits (Qiagen, CA) after standard transformation into DH5α competent bacterial cells. The TAP tagged DEN2 C expression plasmid was made by cloning the 100 amino acid coding region for mature DEN2 C into the N-terminal TAP plasmid (Stratagene, CA). GFP-fusion protein was made by cloning mature DEN2 C into N-GFP-TOPO (Invitrogen, CA) in frame with the N-terminal GFP protein. GST-tagged DEN2 C was made by cloning mature DEN2 C into pGEX-6P-2 (GE, PA) in frame with N-terminal GST tag.

### Transfection of plasmids

All plasmids were transfected into liver cells using Effectene (Qiagen, CA) according to manufacturer's instructions. Briefly, for a 10 cm^2^ plate, 10 µg of DNA was mixed with 500 µL buffer EC and 32 µL enhancer was added. This was allowed to incubate for 5 min on the benchtop. Then, 30 µL Effectene reagent was added and the solution vortexed briefly. After 10 min incubation, the solution was added to the cells. Expression was observed 24 h post-transfection and peaked at 48 h.

### Production of GST-tagged DEN2 C

The pGEX-6P-2-DEN2-C plasmid was transformed into BL-21-Gold (DE3) competent cells (Stratagene, CA) using a standard transformation protocol. The bacteria was grown in LB media overnight at 37°C, diluted in fresh media and grown to mid-log phase (A600 = 0.7). The expression of GST-tagged DEN2 C was induced by adding isopropyl-beta D-thiogalactoside (IPTG) to 0.1 mM final concentration and allowed to grow for 4 hours at 37°C. The cells were sedimented by centrifugation at 10,000 x g and resuspended in PBS(−). The cells were lysed using a French pressure cell press, Triton X-100 was added to a final concentration of 1% and cells were shaken at 25°C for 30 min fo solubilize proteins. The crude extract was centrifuged at 10,000 x g for 5 min at 4°C and the supernatant was incubated with glutathione sepharose (GS) 4B (GE, NJ) for 30 min at 25°C. The GS was centrifuged at 500 x g for 5 min and washed three times with PBS(−). The GST-tagged DEN2 C was eluted by adding elution buffer (50 mM Tris-HCl, 10 mM reduced glutathione, pH 8.0), incubating at 25°C for 10 min and centrifugation at 500 x g for 5 min.

### TAP assay

The TAP assay was used to identify liver cell proteins that interacted with DEN2 C. Briefly, at 48 h post-transfection of NTAP-DEN2-C, Huh7 cells were washed with PBS(−) and lysed using 1X lysis buffer containing protease inhibitors. All steps were done at 4°C to maintain the protein interactions. The cell lysates were applied to streptavidin resin, incubated at 4°C for 2 h, washed and then any bound proteins were eluted off. A second purification step was done with calmodulin resin and proteins boiled off into PBS(−). The eluted proteins were trypsin-digested and LC MS/MS analyzed at the Yale University W.M. Keck Foundation core facility on a LTQ Orbitrap mass spectrometer. All MS/MS spectra were searched using the automated Mascot algorithm against the IPI human database. Identification criteria used was as follows: 2 or more MS/MS spectra match the same protein entry in the database and matched peptides derive from the type of enzymatic digestion performed on the protein.

### Dengue virus infection

Huh7 cells at 80% confluence were infected with dengue 2 New Guinea C virus and human South American isolates of dengue 1, 3 and 4, kind gifts of Dr. John Anderson, CAES, CT (DEN2, DEN1, DEN3, DEN4, respectively) at an MOI of 0.1 or 1.0, as indicated in the figure legends. Virus was added directly to media and left on for 1 h, cells were washed, new media was added and cells were incubated for various timepoints, depending on the assay. Viral stocks were propagated in C6/36 cells for 8 days before supernatant was centrifuged, virus collected and stored at −80°C before use.

### Western blots

The plasmids containing the gene for desired protein expression were transfected into cells and at 48 h the cells were lysed. The lysate was boiled for 5 min in SDS-PAGE buffer and run on a 4–12% SDS-PAGE gel for 1.5 h at 15 milliamps per gel. The proteins were then transferred to nitrocellulose membrane. The nitrocellulose was blocked with 5% milk in 1% TBST for 1 h at RT and then incubated with the appropriate primary antibody overnight at 4°C. The nitrocellulose was washed and then incubated with the appropriate horseradish peroxidase secondary antibody for 1 h at RT. The protein blots were incubated with ECL substrates (Amersham, NJ) for 5 min at RT and then detected on Kodak film. Antibodies used were rabbit polyclonals against histones: H2A, H2B, H3, H4, phosphorylated H2A (ab61250, ab61255, ab18255, ab18521, ab11174, respectively, Abcam, MA), dimethyl H3 (Lys80) (Santa Cruz Tech, CA), the GST tag (ab3416 Abcam, MA) and mouse immune serum against recombinant DEN2 capsid protein made in our laboratory.

### Far Western blots

For the histone probe blots, 20 µg of GST or GST-tagged DEN2 C was run on a 4–12% SDS-PAGE gel and transferred to nitrocellulose as above. The nitrocellulose was incubated with 5 µg of each recombinant histone (H2A, H2B, H3, H4, Active Motif, CA) for 1 h at RT, washed and incubated with primary and secondary antibodies against histones, mentioned above. Proteins were detected as in Western blot methods. For the capsid probe blots, 3 µg of either H2A, H2B, H3, H4, BSA or GFP were run on a 4–12% SDS-PAGE gel and transferred to nitrocellulose. The nitrocellulose was incubated with 10 µg GST-tagged DEN2 C and detected with the GST antibody mentioned above.

### Co-immunoprecipitation

Huh7 cells at 80% confluence were infected with dengue 2 New Guinea C virus at m.o.i of 0.1 and lysed 24 h post-infection. Proteins were separately immunoprecipitated from the lysate using antibodies mentioned in ‘Western blots’ using the Pierce Co-Immunoprecipitation kit (Pierce, IL) according to manufacturer's instructions. Briefly, 400 µg antibody was incubated with coupling resin overnight at RT. Cell lysate was diluted in 200 µL coupling buffer and incubated with antibody-bound resin for 2 h at RT. Resin was washed with coupling buffer three times and then protiens were eluted using 100 µL elution solution. Eluted proteins were run on a 4–12% SDS-PAGE gel and transferred to nitrocellulose. The individual membranes were probed with antibodies or mouse immune serum against DEN2 C protein.

### Immunofluorescence assays

Infected or transfected cells were fixed in 4% paraformaldehyde for 15 min at RT and then permeablilized with 0.1% Triton-X 100 for 1 min at room temperature before incubation with appropriate antibody. Antibodies used were against calmodulin binding protein (CBP) (Abcam, MA), histones: H2A, H2B, H3, H4 (same as previous, Abcam, MA) and mouse immune serum against recombinant DEN2 capsid protein made in our laboratory. Cells were blocked in 1% BSA (Fisher Scientific, PA) in PBS (−) for 20 min at room temperature and were then incubated with primary antibody diluted in 1% BSA 1/250 for 20 minutes at RT. Cells were washed, incubated with labeled secondary antibody diluted in 1% BSA 1/500 for 20 minutes at RT and analyzed by fluorescent microscopy. A DAPI stain (Sigma-Aldrich, MO) was added to the cells for 1 min at RT to illustrate the location of nucleus in the cell.

### Protein binding assays

Recombinant histones H2A, H2B, H3 and H4 were obtained as lyophilized protein (Active Motif, CA) and dissolved in ddH_2_O before use. GST-tagged DEN2 C was produced in E.coli and batch purified using glutathione sepharose (GS) (GE, NJ) with centrifugation. Briefly, pelleted bacteria from 1L culture was lysed and mixed with 2 mL GS resin with end-over-end mixing for 1h at RT. The resin was spun down at 500 rpm, washed with PBS(-) and protein was eluted with buffer (50 mM Tris-HCl, 10 mM reduced glutathione, pH 8.0). The GST-tagged DEN2 C and histones were incubated in binding buffer (10 mM Tris-HCl, pH 7, 5 mM CaCl_2_, 10 mM MgCl_2_) for 1 h at 37°C and analyzed on gels.

### DNA-binding assays

Varying amounts (see figure legend) of recombinant histone and/or DEN2 C proteins were incubated with 300 ng plasmid DNA in binding buffer (20 mM Tris, pH 8.0, 200 mM NaCl) for an hour at 37°C. The solution was run on a 1% TAE agarose gel with ethidium bromide for DNA visualization.

### ELISA analysis

5 µg of GST or GST-tagged DEN2 C was coated onto a 96-well ELISA plate (Thermo Fisher Sci, MA) and incubated overnight at 4°C. The plate was blocked with 1% BSA in PBS(-) and incubated with 1 µg H2A, H2B, H3, H4 or recombinant GFP (Clontech, CA) for an hour at RT. The proteins were washed off, secondary-HRP was added for 30 min at RT, washed off and TMB substrate was added for 20 min at RT. Stop solution was added and the O.D. of the wells read at 450 nm.

### siRNA

The siRNA against H2A and H3 (SMARTpools M-011682-01 and M-011684-01 respectively, Dharmacon, CO) were transfected using DharmaFECT4 according to manufacturer's instructions (Dharmacon, CO). Briefly, Huh-7 cells were grown to confluence in a 6-well tissue culture plate. The siRNA solution was prepared using 2 eppendorf tubes in the tissue culture hood. In tube 1, 100 µL 2 µM siRNA was added to 100 µL serum-free DMEM medium. In tube 2, 3 µL DharmaFECT4 reagent was added to 200 µL serum-free DMEM medium. Both tubes were allowed to incubate at RT before mixing and incubating the solution at RT for 20 minutes. Then, 500 µL complete medium was added and the entire solution was added to cells with no media present.

### QPCR

RNA was isolated from cell extracts using Rneasy kits (Qiagen, CA). cDNA was made from the RNA using the SuperscriptIII kit (Invitrogen, CA). An aligquot of cDNA made from 1 µg RNA was used in each quantitative (q)PCR reaction. Primers were as follows: dengue envelope: 5′-caggctgaggatggacaaactac-3′ & 5′-caaaagggatcttacatggagaac-3′, GAPDH: 5′-gaaggtgaaggtcggagtc-3′ & 5′-gaagatggtgatgggatttc-3′.

## Supporting Information

Figure S1 **Expression of DENV C in NTAP vector.** The 100 amino acid mature DENV C protein fused to streptavidin binding protein and calmodulin binding protein (CBP) tags was expressed in Huh7 liver cells. Cells were fixed with 4% paraformaldehyde and stained with an antibody against CBP tag. Cells were counterstained with DAPI for nucleus visualization.
(TIFF)

Figure S2 **Expression of DENV C-GFP fusion protein.** Huh7 cells were transfected with DENV C-GFP fusion (A) plasmid or an expression plasmid coding for GFP alone (B). Cells were fixed with 4% PFA at 24 h post-transfection. GFP-DENV-C localizes to the nucleus and expression changes cell morphology when compared to expression of GFP alone.
(TIFF)

Figure S3 **Structural similarity between capsid and histone H2A.** (A) A ribbon diagram of H2A closely resembles (B) a ribbon diagram of capsid protein, with 4 alpha-helices each. The 2nd alpha-helix is designated. EsyPred3D software was used to make the diagrams. (C and D) The secondary structure of H2A (C) and DENV C (D) with labeled alpha-helices. Hydrophobic amino acids in the 2nd alpha-helix that likely contribute to heterodimerization are in red.
(TIFF)

Figure S4 **DENV infection does not alter H3 dimethylation.** Huh7 cells were transfected with DENV C and/or infected with DENV 2 NGC 24 h post-transfection. Cells were lysed 24 h post-infection (48 h post-transfection) and lysates were run on 4–12% SDS-PAGE gel. Gels were used in a Western blotting assay with antibodies that detect H3 and dimethylated lysine 80 of histone H3. Gels were stripped and reprobed with an antibody against actin as loading control.
(TIFF)
