# Supplementary figures and images for: Dengue Virus Capsid Protein Binds Core Histones and Inhibits Nucleosome Formation in Human Liver Cells
Source: PLoS One. 2011 Sep 1;6(9):e24365. doi: 10.1371/journal.pone.0024365 (PMC3164731; doi:10.1371/journal.pone.0024365)

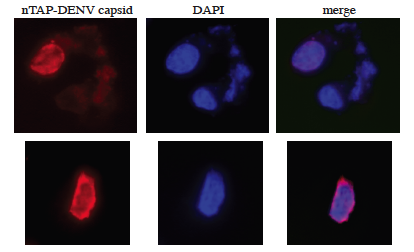

Supplement: Figure S1 — Expression of DENV C in NTAP vector. The 100 amino acid mature DENV C protein fused to streptavidin binding protein and calmodulin binding protein (CBP) tags was expressed in Huh7 liver cells. Cells were fixed with 4% paraformaldehyde and stained with an antibody against CBP tag. Cells were counterstained with DAPI for nucleus visualization. (TIFF) [file pone.0024365.s001.tiff]

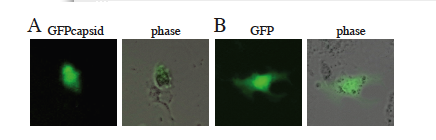

Supplement: Figure S2 — Expression of DENV C-GFP fusion protein. Huh7 cells were transfected with DENV C-GFP fusion (A) plasmid or an expression plasmid coding for GFP alone (B). Cells were fixed with 4% PFA at 24 h post-transfection. GFP-DENV-C localizes to the nucleus and expression changes cell morphology when compared to expression of GFP alone. (TIFF) [file pone.0024365.s002.tiff]

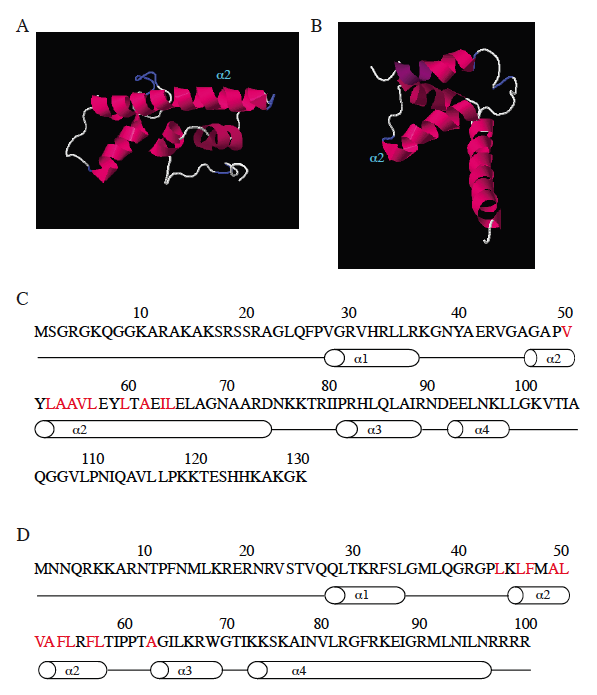

Supplement: Figure S3 — Structural similarity between capsid and histone H2A. (A) A ribbon diagram of H2A closely resembles (B) a ribbon diagram of capsid protein, with 4 alpha-helices each. The 2nd alpha-helix is designated. EsyPred3D software was used to make the diagrams. (C and D) The secondary structure of H2A (C) and DENV C (D) with labeled alpha-helices. Hydrophobic amino acids in the 2nd alpha-helix that likely contribute to heterodimerization are in red. (TIFF) [file pone.0024365.s003.tiff]

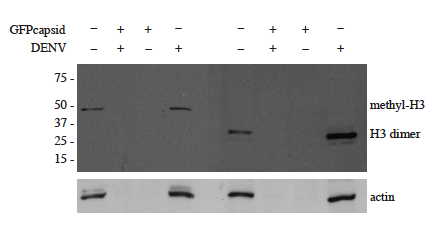

Supplement: Figure S4 — DENV infection does not alter H3 dimethylation. Huh7 cells were transfected with DENV C and/or infected with DENV 2 NGC 24 h post-transfection. Cells were lysed 24 h post-infection (48 h post-transfection) and lysates were run on 4–12% SDS-PAGE gel. Gels were used in a Western blotting assay with antibodies that detect H3 and dimethylated lysine 80 of histone H3. Gels were stripped and reprobed with an antibody against actin as loading control. (TIFF) [file pone.0024365.s004.tiff]
